# PD-L1 Is a Tumor Suppressor in Aggressive Endometrial Cancer Cells and Its Expression Is Regulated by miR-216a and lncRNA MEG3

**DOI:** 10.3389/fcell.2020.598205

**Published:** 2020-12-09

**Authors:** Daozhi Xu, Peixin Dong, Ying Xiong, Rui Chen, Yosuke Konno, Kei Ihira, Junming Yue, Hidemichi Watari

**Affiliations:** ^1^Department of Obstetrics and Gynecology, Hokkaido University School of Medicine, Hokkaido University, Sapporo, Japan; ^2^Department of Gynecology, State Key Laboratory of Oncology in South China, Sun Yat-sen University Cancer Center, Guangzhou, China; ^3^Department of Pathology and Laboratory Medicine, University of Tennessee Health Science Center, Memphis, TN, United States; ^4^Center for Cancer Research, University of Tennessee Health Science Center, Memphis, TN, United States

**Keywords:** PD-L1, MEG3, miR-216a, long non-coding RNA, EMT, endometrial cancer

## Abstract

**Background:**

Poorly differentiated endometrioid adenocarcinoma and serous adenocarcinoma represent an aggressive subtype of endometrial cancer (EC). Programmed death-ligand-1 (PD-L1) was known to exhibit a tumor cell-intrinsic function in mediating immune-independent tumor progression. However, the functional relevance of tumor cell-intrinsic PD-L1 expression in aggressive EC cells and the mechanisms regulating its expression remain unknown.

**Methods:**

PD-L1 expression in 65 EC tissues and 18 normal endometrium samples was analyzed using immunohistochemical staining. The effects of PD-L1 on aggressive EC cell growth, migration and invasion were investigated by cell functional assays. Luciferase reporter assays were used to reveal the microRNA-216a (miR-216a)-dependent mechanism modulating the expression of PD-L1.

**Results:**

Positive PD-L1 expression was identified in 84% of benign cases but only in 12% of the EC samples, and the staining levels of PD-L1 in EC tissues were significantly lower than those in the normal tissues. Higher PD-L1 expression predicts favorable survival in EC. Ectopic expression of PD-L1 in aggressive EC cells results in decreased cell proliferation and the loss of mesenchymal phenotypes. Mechanistically, PD-L1 exerts the anti-tumor effects by downregulating MCL-1 expression. We found that PD-L1 levels in aggressive EC cells are regulated by miR-216a, which directly targets *PD-L1*. We further identified a mechanism whereby the long non-coding RNA MEG3 represses the expression of miR-216a, thereby leading to increased PD-L1 expression and significant inhibition of cell migration and invasion.

**Conclusion:**

These results reveal an unappreciated tumor cell-intrinsic role for PD-L1 as a tumor suppressor in aggressive EC cells, and identify MEG3 and miR-216a as upstream regulators of PD-L1.

## Introduction

Endometrial cancer (EC) is the most common gynecologic malignancy in developed countries, with approximately 89,929 deaths worldwide in 2018 (Bray et al., 2018). ECs are classified into various histological subtypes: including endometrioid EC, serous EC, clear-cell EC, and mixed (usually endometrioid and serous components) EC (Gaber et al., 2016). Unlike the majority of ECs, which are usually associated with well-differentiated endometrioid histology, early stage disease, and a more favorable prognosis, poorly differentiated endometrioid ECs and serous ECs are generally seen in older patients, are more aggressive, and are characterized by a high rate of recurrence and metastasis (Gaber et al., 2016).

In the early stage of tumor metastasis, an epithelial-mesenchymal transition (EMT) program occurs and contributes to the acquisition of invasive properties, enabling epithelial cells to lose polarity and adhesion capacity, but acquire the features of mesenchymal cells (Valastyan and Weinberg, 2011). A small cohort of transcription factors (such as TWIST1, ZEB1, Snail, and Slug) is recognized to affect EMT induction by controlling the expression of epithelial genes (for example, ZO-1 and E-cadherin) and mesenchymal genes (including Vimentin and HSP47) (Valastyan and Weinberg, 2011). MCL-1, a member of the Bcl-2 family of proteins, plays an essential role in promoting cell survival and metastasis in many cancers (De Blasio et al., 2018) and was discovered to be the key EMT inducer in EC (Konno et al., 2014). The EMT process is also regulated by complex epigenetic regulatory mechanisms, such as DNA methylation and histone modifications, as well as non-coding RNAs, including microRNAs (miRNAs) and long non-coding RNAs (lncRNAs) (Kiesslich et al., 2013; Xu et al., 2020). However, the molecular mechanisms governing EMT in aggressive EC remain poorly understood.

Evasion of the immune system is classified as a hallmark of cancer, which allows cancer cells to escape the attack from immune cells (Hanahan and Weinberg, 2011). Tumor cells can exploit the programmed death-1 (PD-1) immune checkpoint pathway to evade immune cells (Okazaki and Honjo, 2007). Programmed death-ligand-1 (PD-L1) is a critical immune checkpoint ligand expressed on the surface of tumor cells, and binding of PD-L1 to its receptor PD-1 on activated T cells inhibits anti-tumor immunity by reducing the proliferation, cytokine secretion, and cytotoxic ability of T cells (Okazaki and Honjo, 2007). Despite this well-characterized function of PD-L1 in cancer, emerging studies demonstrate a tumor cell-intrinsic function of PD-L1 in mediating EMT and immune-independent tumor progression (Dong et al., 2018c). Elevated expression levels of PD-L1 were detected in diverse tumors, and PD-L1 upregulation was significantly correlated with poor survival (Dong et al., 2018c). For example, PD-L1 was highly expressed in cervical cancer tissues, and overexpression of PD-L1 significantly increased cancer cell proliferation and invasion (Dong et al., 2018a). However, the silencing of PD-L1 expression in cholangiocarcinoma cells and lung cancer cells promoted tumor growth (Tamai et al., 2014; Wang X. et al., 2020). In patients with melanoma (Taube et al., 2012), colorectal cancer (Droeser et al., 2013), or Merkel-cell carcinoma (Lipson et al., 2013), higher PD-L1 expression was significantly correlated with improved survival, indicating that tumor cell-intrinsic PD-L1 may exert either pro- or anti-tumor functions, and that its roles might be tissue- or tumor-type dependent.

Previous studies have shown conflicting results regarding the expression patterns of PD-L1 and its prognostic value in EC (Marinelli et al., 2019). The fraction of ECs that are positive for PD-L1 expression varied from 14 to 59% (Mo et al., 2016; Li et al., 2018; Engerud et al., 2020). Although it was reported that PD-L1 expression has no significant impact on patient survival (Engerud et al., 2020), recent studies presented contrary results showing that higher PD-L1 expression in EC samples was associated with improved survival (Yamashita et al., 2017; Liu et al., 2019; Zhang et al., 2020). Also, increased PD-L1 expression in EC cells was significantly correlated with well-differentiated histology and a lower risk of myometrial invasion and lymphatic spread (Zhang et al., 2020).

Until now, the biological function of PD-L1 and the mechanisms associated with PD-L1 expression in aggressive EC cells have not yet been explored. In this study, we found that PD-L1 protein expression was frequently downregulated in EC tissues and that increased PD-L1 expression level was associated with prolonged survival rates of EC patients. Our data identified PD-L1 as a novel tumor suppressor in aggressive EC cells and showed that loss of PD-L1 expression enhances the invasive abilities of EC cells by inducing EMT through the upregulation of MCL-1 expression. We further define a molecular mechanism whereby the long non-coding RNA MEG3/microRNA-216a (miR-216a) axis mediates the repression of PD-L1 in aggressive EC cells. Thus, this work uncovered a unique, tumor-suppressor function of PD-L1 in an aggressive subtype of EC, where options for effective treatment are limited.

## Materials and Methods

### Patients and Tissue Specimens

Acquisition and use of human tissue samples was approved by the Research Medical Ethics Committee of Sun Yat-sen University Cancer Center. Written consent was obtained from each patient before sample collection. A total of 65 human primary EC tissues and 18 normal endometrium samples were obtained from the patients who underwent surgical resection without preoperative treatment, at the Sun Yat-sen University Cancer Center. The diagnosis of EC or normal endometrium was confirmed by histological examination. All clinical and pathological features, including tumor grade, tumor stage, tumor size, and myometrial invasion, were obtained from hospital records.

### Immunohistochemistry of Human Tissues

Immunohistochemical (IHC) staining was performed as we previously described (Konno et al., 2014). In brief, formalin-fixed, paraffin-embedded tissues of 65 primary EC tissues and 18 normal endometrium tissues were analyzed for the expression of PD-L1. Sections from these tissues were deparaffinized in xylene, rehydrated in grades of alcohol, rinsed in tap water, and blocked with 3% hydrogen peroxide. After antigen retrieval by citrate buffer using a microwave oven, the sections were incubated with the primary antibody against PD-L1 (dilution 1:200, clone E1L3N, Cell Signaling, Danvers, MA, United States) at 4°C overnight. For positive control, this study used human cervical cancer tissues with previously characterized levels of PD-L1 expression (Dong et al., 2018a). Negative control slides without primary antibodies were also included. The IHC staining score was assessed by two experienced pathologists who were blinded to the patients’ clinicopathological data. For the purpose of this study, the stromal tissue was excluded from scoring. Scoring was based on intensity and extensity. The percentage of stained cells was determined as: 0 (no positive cell), 1 (<10%), 2 (10–50%), and 3 (>50%). The intensity of IHC staining was determined as: 0 (no staining), 1 (weak staining), 2 (moderate staining), and 3 (strong staining). The overall IHC score of each section was calculated by adding the proportion score to the intensity score of each case (range, 0–6), as previously reported (Konno et al., 2014). Any sample was defined as having positive PD-L1 staining if the overall IHC score was ≥1, and any sample was defined as having negative PD-L1 staining if the overall IHC score was 0. Overall IHC scores of ≥2 were defined as a high expression of PD-L1 staining, and overall IHC scores of <2 were defined as a low expression of PD-L1 staining, based on receiver operating characteristic (ROC) analysis.

### Human Cell Lines and Cell Culture

Human EC cell lines, including HEC-50 (JCRB Cell Bank, Osaka, Japan) and HOUA-I (RIKEN Cell Bank, Tsukuba, Japan), were derived from poorly differentiated endometrioid EC. The human EC cell line HEC-1 (JCRB Cell Bank, Osaka, Japan) was derived from a moderately differentiated endometrioid EC and has invasive properties. The highly invasive sub-population of HEC-50 cells (referred to as HEC-50-HI cells) was generated using Matrigel invasion chambers, as we previously described (Dong et al., 2011). The immortalized human endometrial epithelial cell line (EM) was a kind gift from Dr. Satoru Kyo (Shimane University, Japan) (Kyo et al., 2003). The human cervical cancer cell line HeLa was obtained from the American Type Culture Collection (ATCC). These cells were cultured in DMEM/F12 medium (Sigma-Aldrich, St. Louis, MO, United States) supplemented with 10% fetal bovine serum (FBS) (Invitrogen, Carlsbad, CA, United States). The human serous EC cell line SPAC-1-L was kindly provided by Dr. Fumihiko Suzuki (Tohoku University, Sendai, Japan) and maintained in RPMI-1640 medium (Sigma-Aldrich, St. Louis, MO, United States) supplemented with 10% FBS (Invitrogen).

### Transient Transfection

The *PD-L1* cDNA expresion vector pCMV6-PD-L1 (PD-L1-vec, RC213071), the *MCL-1* cDNA expression vector pCMV6-MCL-1 (MCL-1-vec, RC200521), the MEG3 expression vector pCMV6-MEG3 (MEG3-vec, SC105816) and the pCMV6 control vector (Ctr-vec, PS100001) were purchased from OriGene (Rockville, MD, United States). The *PD-L1*-specific siRNA (PD-L1-siRNA, s26547), the *MCL-1*-specific siRNA (MCL-1-siRNA, AM51331), the MEG3-specific siRNA (MEG3-siRNA, n272552), the negative control siRNA (Ctr-siRNA, AM4611), miR-216a mimic (PM10545), control mimic (AM17110), miR-216a inhibitor (AM10545) and control inhibitor (AM17010) were purchased from Invitrogen (Carlsbad). The cells were seeded in growth medium at a density of 40–50% 1 day before transfection. Transfection of vector, siRNA, miRNA mimics or miRNA inhibitor into EC cells was carried out using the Lipofectamine 2000 reagent (Invitrogen) following the manufacturer’s instructions. After 48 h, the cells were harvested for the following tests.

### Western Blotting

Protein extracts for western blotting were prepared in M-Per Mammalian Protein Extraction Reagent (Pierce, Rockford, IL, United States), separated by SDS-polyacrylamide gels, and then transferred to PVDF membrane (GE Healthcare Life Sciences, Piscataway, NJ, United States). Membranes were incubated with primary antibodies including: PD-L1 (1:1000, clone E1L3N, Cell Signaling), MCL-1 (1:1000, #4572, Cell Signaling), ZO-1 (1:1000, #5406, Cell Signaling), Vimentin (1:1000, A01189, GenScript, Edison, NJ, United States), and GAPDH (1:3000, sc-47724, Santa Cruz Biotechnologies, Santa Cruz, CA, United States), and then with HRP-conjugated secondary antibody. Finally, blots were developed with the ECL detection kit (Amersham Pharmacia Biotech, United Kingdom). GAPDH served as the loading control. Immunoblot images were quantified using the NIH Image software.

### PD-L1 Knockdown and Overexpression in EC Cells

To silence *PD-L1* gene expression, lentiviral particles encoding two short hairpin RNA RNAs (shRNAs: HSH064502 and HSH099746) targeting *PD-L1* and a control shRNA (CSHCTR001) were purchased from Genecopoeia (Guangzhou, China). Stably transfected HEC-50 cells were selected using 1 μg/mL puromycin (Sigma-Aldrich, St. Louis, MO, United States). For overexpressing PD-L1 in SPAC-1-L cells, PD-L1-vec and Ctr-vec were used to transfect SPAC-1-L cells using the Lipofectamine 2000 reagent (Invitrogen). Stable PD-L1-overexpressing SPAC-1-L cells and control cells were selected using 0.5 mg/mL neomycin (Sigma-Aldrich, St. Louis, MO, United States) and confirmed by western blotting for PD-L1.

### Cell Proliferation Assay

Cell proliferation was investigated using the Cell Counting Kit-8 (CCK-8) assay (Dojindo, Japan) according to the manufacturer’s instructions. Five thousand cells were seeded per well in a 96-well plate and cultured for the indicated times. 10 μl of CCK-8 reagent was added into each well and incubated for 1 h. The absorbance was assessed at 450 nm by a microplate reader (Bio-Rad, Hercules, CA, United States). Each experiment was performed in triplicate.

### Wound-Healing Assay

Wound-healing assay was performed as previously described (Konno et al., 2014). In brief, confluent cells were scraped by a 200 μl pipette tip to create a wound, and debris was removed by PBS washing. Growth media were replaced with fresh media containing Mitomycin C (5 μg/ml, Sigma-Aldrich, St. Louis, MO, United States) and incubated for 12 h. Cells were imaged after creation of the wound and 12 h later. Distance migrated was quantitated by taking pictures at 0 and 12 h.

### Transwell Invasion Assay and Transwell Migration Assay

For invasion assays, 5 × 10^4^ cells suspended in medium without FBS were plated on the upper wells of Matrigel-coated Transwell plates (8 μm pore size, Corning Costar Co., Lowell, CA, United States). The insert was incubated in 750 μl medium with 10% FBS. After culturing for 24 h, the membranes were treated with 10% formaldehyde for 3 min, and then stained with 2% crystal violet for 15 min at room temperature. The non-invasive cells were removed by swiping the top of membrane with cotton swabs. Cells that invaded across the transwell membrane were counted using a light microscope in 10 randomly selected high-power fields. Transwell migration assays were performed in the same manner as the transwell invasion assays, except that the membrane was not coated with Matrigel, and the incubation time was 12 h.

### Caspase-Glo 3/7 Assay

The enzymatic activity of caspase-3/7 was determined using the Caspase-Glo 3/7 assay kit following the manufacturer’s instructions (Promega, Madison, WI, United States), as previously reported (Konno et al., 2014). Briefly, 3 × 10^3^ cells were plated in triplicates in 96-well plates and transfected as indicated. An equal amount of Caspase-Glo 3/7 substrate was added to the culture and subsequently incubated for 3 h. Following incubation, caspase-3/7 activities were evaluated as an indicator of cell apoptosis using a GloMax-96 Microplate luminometer (Promega). The results were shown as the fold change relative to the control cells.

### Gain-of-Function Screening for PD-L1 Based on Cell Functional Assays

The indicated EC cell lines or human cervical cancer cell line HeLa were transiently transfected with PD-L1-vec or Ctr-vec using the Lipofectamine 2000 reagent (Invitrogen). After incubation for 48 h, cell proliferation, apoptosis, migration, and invasion were determined using CCK-8 assay, Caspase-Glo 3/7 assay, transwell migration assay, and transwell invasion assay, respectively. The results were given as the fold changes in cell proliferation, apoptosis, migration and invasion of the PD-L1-vec-transfected cells as compared to the Ctr-vec-transfected cells.

### Quantitative Reverse Transcription-PCR (qRT-PCR)

Total RNA was isolated with the TRIzol reagent (Invitrogen). Total mRNA was reversely transcribed into cDNA using an M-MLV Reverse Transcriptase Kit (Invitrogen). Quantitative RT-PCR was performed using SYBR Premix Ex Taq II (Takara, Shiga, Japan) in an ABI-7300 Real-Time PCR system (Applied Biosystems, Foster City, CA, United States). The primers used were as follows: human *PD-L1*, sense: GTGGC ATCCAAGATACAAACTCAA, anti-sense: TCCTTCCTCTTG TCACGCTCA; human *ZO-1*, sense: GGAGAGGTGTTCCG TGTTGT, anti-sense: GAGCGGACAAATCCTCTCTG; human *Vimentin*, sense: TGAGTACCGGAGACAGGTGCAG, anti-sense: TAGCAGCTTCAACGGCAAAGTTC; human *Snail*, sense: GACCACTATGCCGCGCTCTT, anti-sense: TCGCTy GTAGTTAGGCTTCCGATT; human *MCL-1*, sense: CCAAGG CATGCTTCGGAAA, anti-sense: TCACAATCCTGCCCCAG TTT; human MEG3, sense: TCCATGCTGAGCTGCTGCCAAG, anti-sense: AGTCGACAAAGACTGACACCC; and human *GAPDH*, sense: GAAGGTGAAGGTCGGAGTC, anti-sense: GAAGATGGTGATGGGATTTC. *GAPDH* was used as an internal control. The levels of miR-138/193a/216a/217 were measured using the NCode SYBR GreenER miRNA qRT-PCR analysis kit (Invitrogen) according to the manufacturer’s protocol. The forward primers for miRNA analysis had the same sequences as the mature miRNAs. The relative expression of these miRNAs was normalized against that of the U6 endogenous control.

### Luciferase Reporter Assay

Human *PD-L1* 3′-untranslated region (3′-UTR) luciferase reporter vector and the Luc-Pair^TM^ Duo-Luciferase Assay Kit were purchased from GeneCopoeia (Rockville, MD, United States). Mutations (MUT) of the miR-216a binding site in the *PD-L1* 3′-UTR were generated using a QuickChange site-directed mutagenesis kit (Stratagene, La Jolla, CA, United States). EC cells with 60–70% confluency were co-transfected with the wild-type (WT) or mutant *PD-L1* 3′-UTR luciferase reporter vector, together with miR-216a mimic, miR-216a inhibitor, or the respective control, using the Lipofectamine 2000 reagent (Invitrogen). 48 h later, cell lysates were collected and the Firefly and Renilla luciferase activities of each group were tested following the protocol of the Luc-Pair^TM^ Duo-Luciferase Assay Kit (GeneCopoeia). The Firefly luciferase activities were normalized by Renilla luciferase activities.

### Statistical Analysis

All experiments were carried out with at least three replicates. Data are presented as the mean ± standard error of the mean. Statistical analysis was performed using SPSS 18.0 statistical software (SPSS, Chicago). Comparisons between two groups were made using the two-tailed Student’s *t*-tests and Mann–Whitney *U* tests. The χ^2^-tests and Fisher’s exact tests were applied to analyze the relationship between PD-L1 expression and clinicopathological status. Differences were considered statistically significant when *P* < 0.05.

## Results

### Downregulation of PD-L1 Correlates With Poor Survival in EC

**FIGURE 1:**
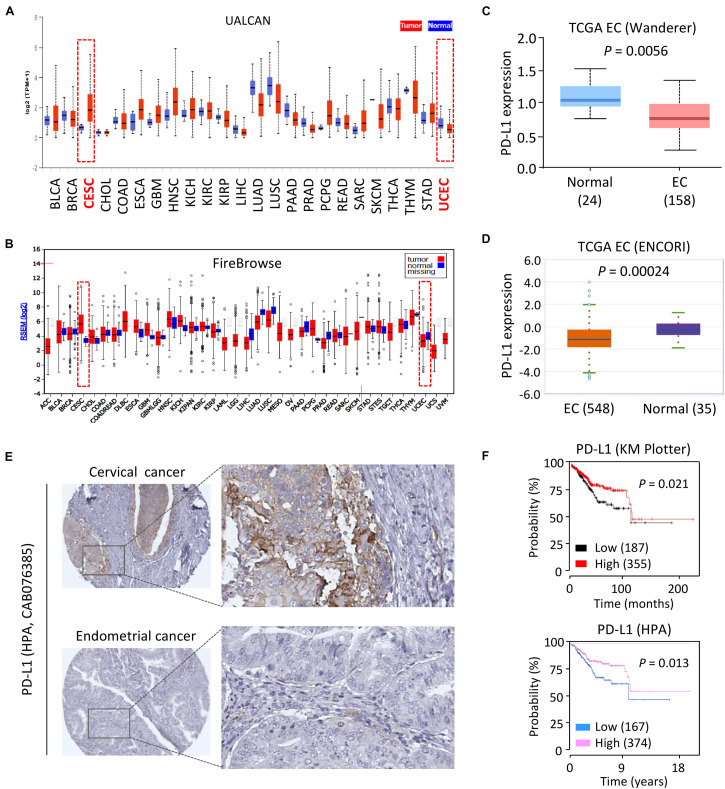
Downregulation of PD-L1 Correlates with Poor Survival in EC. **(A,B)** Analysis of PD-L1 expression in human cancer tissues and normal tissues using the TCGA data retrieved from the UALCAN database **(A)**, and FireBrowse database **(B)**. CESC: human cervical cancer; UCEC: human endometrial cancer. **(C,D)** PD-L1 mRNA expression in EC samples and normal samples was analyzed using the Wanderer database **(C)** and ENCORI database **(D)**. **(E)** IHC staining for PD-L1 in human cervical cancer tissues and EC tissues with the same antibody (CAB076385, the HPA website) showed that high levels of PD-L1 were present in primary cervical cancer tissues. In contrast, PD-L1 was negatively or weakly stained in most EC tissues. **(F)** The probability of overall survival in EC patients expressing high or low PD-L1 levels was assessed using the KM plotter database (upper) and the HPA database (bottom). Red indicates CESC (human cervical cancer) or UCEC (human endometrial cancer).

First, the mRNA expression of *PD-L1* in different cancer types was assessed by comparing normal vs. tumor tissues using the UALCAN portal, which contains the Cancer Genome Atlas (TCGA) gene expression data from 31 cancer types for further analysis (Chandrashekar et al., 2017). Consistent with our previous study (Dong et al., 2018a), we observed relatively higher levels of PD-L1 in human cervical cancer tissues as compared to normal tissues (Figure 1A). Interestingly, the mRNA expression of *PD-L1* in primary EC tissues was lower than that in the normal samples (Figure 1A). To verify these results, we examined the expression of *PD-L1* in the TCGA datasets at the Firebrowse website (Deng et al., 2017). The results revealed that *PD-L1* expression was clearly elevated in cervical cancer tissues compared with normal tissues (Figure 1B). However, EC tissues showed a decrease in PD-L1 levels as compared to normal tissues (Figure 1B). Similarly, the comparison of PD-L1 expression in EC tissues vs. normal tissues though the Wanderer and ENCORI databases (Li et al., 2014; Díez-Villanueva et al., 2015) demonstrated significantly lower expression of PD-L1 in EC tissues (Figures 1C,D).

To determine whether PD-L1 protein expression was also differentially regulated in EC tissues, we extracted the IHC images from the Human Protein Atlas (HPA) database (Uhlén et al., 2015). As expected, PD-L1 was expressed at high levels in human cervical cancer tissues but not in the adjacent normal tissues (Figure 1E). In contrast, IHC staining of EC tissues and adjacent normal tissues with the same antibody showed that PD-L1 levels are very low or completely absent in most EC cells (Figure 1E).

To establish the prognostic importance of PD-L1 expression in EC, we visited the KM plotter (Nagy et al., 2018) and HPA databases to analyze the effects of PD-L1 expression in patients with EC. Higher expression of PD-L1 was associated with increased overall survival in EC patients (Figure 1F). When individual tumor grades (1, 2, and 3) were considered, higher PD-L1 represented a favorable factor for the prognosis of EC patients at histological grade 3 (*P* = 0.014) (Supplementary Figure 1). There was a marginally significant relationship between increased PD-L1 expression and improved overall survival in patients at histological grade 2 (*P* = 0.073) (Supplementary Figure 1). There was no association of PD-L1 expression with survival in EC patients at histological grade 1 (data not shown). These results suggested that increased PD-L1 expression predicts favorable survival in EC (practically those patients with high-grade disease).

### The Protein Expression of PD-L1 Is Downregulated in Human EC Tissues, and PD-L1 Acts as a Tumor Suppressor in Aggressive EC Cells

To further explore the expression of PD-L1 in EC, we examined PD-L1 protein expression in 65 primary EC tissues and 18 normal endometrium tissues using IHC analysis. For our study, we used a rabbit monoclonal antibody (clone E1L3N, Cell Signaling), which is highly sensitive and specific for the detection of PD-L1 protein (Kluger et al., 2017; Keller et al., 2018), and which was validated in our previous IHC study (Dong et al., 2018a).

**FIGURE 2:**
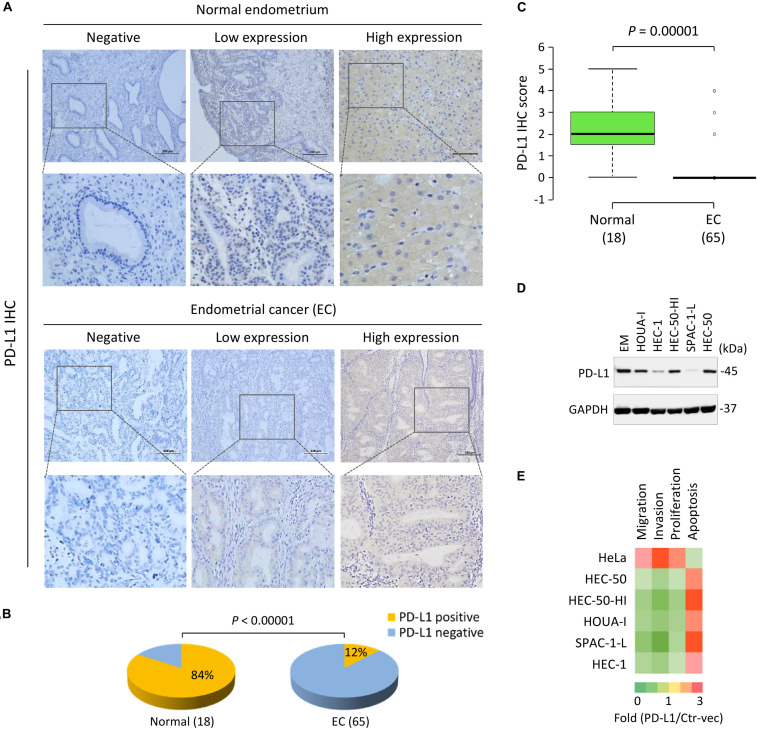
PD-L1 is Frequently Downregulated in EC Tissues and Acts as a Tumor Suppressor in Aggressive EC Cells. **(A)** Representative immunohistochemical staining (IHC) images of PD-L1 in EC tissues and normal endometrium tissues. Scale bar, 200 μm. PD-L1 staining was mostly detected in cell cytoplasm and membrane. **(B)** The pie chart showing the percentages of the population with PD-L1-negative or PD-L1-positive expression in EC tissues and normal endometrium tissues, determined by IHC analysis. **(C)** The mean PD-L1 staining score in EC tissues and normal tissues. The mean PD-L1 staining score in EC tissues was significantly lower than that in the normal tissues. **(D)** PD-L1 protein levels in a normal endometrial cell line (EM) and human aggressive EC cell lines were determined by western blot analysis. **(E)** A gain-of-function screening for PD-L1 that affects the proliferation, apoptosis, migration and invasion of aggressive EC cells. The indicated EC cell lines or human cervical cancer cell line HeLa were transiently transfected with PD-L1 expression vector (PD-L1-vec) or the control vector (Ctr-vec), and subjected to cell functional assays. Heatmap depicting the fold changes in cell proliferation, apoptosis, migration and invasion of the PD-L1-vec-transfected cells when compared with the Ctr-vec-transfected cells.

The positive PD-L1 staining in EC cells was localized mainly in the cytoplasm, although some membranous localization was seen (Figure 2A). As illustrated in Figure 2B, PD-L1 was broadly expressed in normal tissues; however, PD-L1 expression was absent or low in EC tissues. The positive staining rate of PD-L1 was 84% in benign samples, but only 12% in EC samples (*P* < 0.00001) (Figure 2B). Moreover, the mean PD-L1 staining score in EC tissues was significantly lower than that in normal tissues (*P* = 0.00001) (Figure 2C). A trend of higher PD-L1 expression was noted in patients younger than 50 years, and in patients with low-grade tumors (grade 1/2), early stage tumors (stage I), smaller tumor size (tumor diameter ≤3 cm), or superficial myometrial invasion (≤1/2), although this did not reach statistical significance (*P* > 0.05) (Supplementary Figure 2).

Consistent with the immunocytochemistry results, our western blotting analysis confirmed that PD-L1 protein was expressed at lower levels in all aggressive EC cell lines compared to a normal endometrial cell line (EM) (Figure 2D). In particular, PD-L1 expression in the serous EC cell line SPAC-1-L was much weaker than that in other EC cells (Figure 2D and Supplementary Figure 3A). HEC-50-HI cells, the sub-population of HEC-50 cells, are more invasive than the parental HEC-50 cells and exhibit mesenchymal phenotypes (Dong et al., 2011). Interestingly, HEC-50-HI cells showed lower levels of PD-L1 expression than the parental cells (Figure 2D), indicating that PD-L1 may modulate the invasive properties of aggressive EC cells.

To examine this possibility, we transfected the human *PD-L1* cDNA expression vector or the control vector into five aggressive EC cell lines and cervical cancer cell line HeLa for 48 h, and performed a gain-of-function screening using cell functional assays. Consistent with our previous observations (Dong et al., 2018a), we found that the overexpression of PD-L1 could significantly enhance the proliferation, migration, and invasion, but reduce apoptosis of HeLa cells (Figure 2E). Our functional screening revealed that overexpressing PD-L1 expression in all aggressive EC cells significantly attenuated cell proliferation, migration, and invasion, while inducing cell apoptosis (Figure 2E). Taken together, these results indicated that loss of PD-L1 protein expression is a frequent event in EC, and that PD-L1 plays tumor-suppressive roles in aggressive EC cells.

### Loss of PD-L1 Induces Cell Proliferation and Triggers EMT in Aggressive EC Cells

**FIGURE 3:**
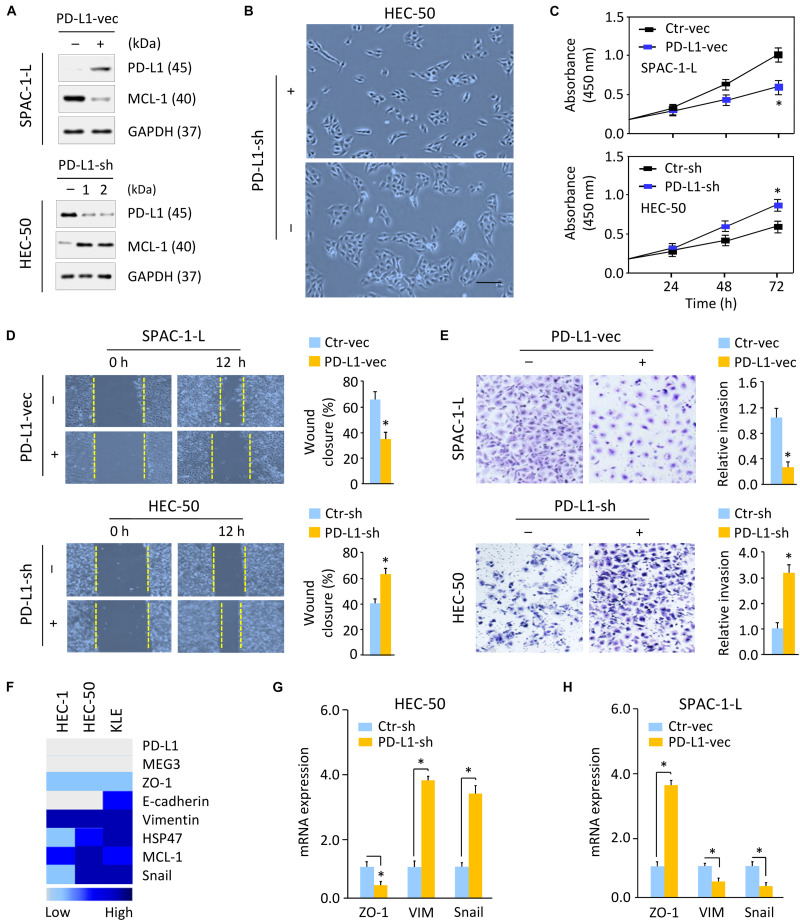
Loss of PD-L1 Induces Cell proliferation and Triggers EMT in Aggressive EC Cells. **(A)** Western blotting analysis of PD-L1 and MCL-1 expression in SPAC-1-L cells overexpressing PD-L1, and in PD-L1-silenced HEC-50 cells. **(B)** Cellular morphology of HEC-50 cells after knockdown of PD-L1. Scale bar, 100 μm. **(C–E)** Proliferation **(C)**, wound-healing **(D)**, and invasion **(E)** assays in EC cells after overexpression or knockdown of PD-L1. **(F)** A heatmap showing gene expression levels in human aggressive EC cell lines (Expression Atlas database). **(G,H)** Examination of gene expression in HEC-50 cells after knockdown of PD-L1 **(G)** and in SPAC-1-L cells after overexpression of PD-L1 **(H)** was performed using qRT-PCR assays. VIM, Vimentin. ^∗^*P* < 0.05.

Next, we investigated whether decreased PD-L1 expression is required for enhanced aggressive EC cell proliferation and invasiveness. We established SPAC-1-L cells stably overexpressing PD-L1 and control cells (Figure 3A and Supplementary Figure 3B), as well as HEC-50 cells stably expressing control shRNA, or two PD-L1 shRNAs (PD-L1-sh-1 or PD-L1-sh-2) that silenced PD-L1 expression (Figure 3A and Supplementary Figure 3B). Because PD-L1-sh-2 showed better performance than PD-L1-sh-1, we used PD-L1-sh-2 for all the downstream experiments.

Although SPAC-1-L cells are invasive, this cell displays an epithelial-like morphology. Overexpression of PD-L1 did not influence the appearance of SPAC-1-L cells (data not shown). However, PD-L1 knockdown in HEC-50 cells induced cell scattering (Figure 3B). Compared to control cells, overexpression of PD-L1 significantly decreased the proliferative and invasive capabilities of SPAC-1-L and HEC-50 cells, while silencing of PD-L1 in HEC-50 and SPAC-1-L cells significantly enhanced these abilities (Figures 3C–E and Supplementary Figures 4A–C). These results indicated that EMT may be involved in the PD-L1-suppressed migratory and invasive abilities of aggressive EC cells.

Using the Expression Atlas database (Papatheodorou et al., 2020), which includes RNA sequencing datasets from human cancer cell lines, we generated a heatmap showing the expression levels of *PD-L1*, EMT regulators (*MCL-1* and *Snail*), epithelial markers (*E-cadherin* and *ZO-1*) and mesenchymal markers (*Vimentin* and *HSP47*) in three aggressive EC cell lines (HEC-1, HEC-50, and KLE). These cells expressed low levels of *PD-L1*, *E-cadherin*, and *ZO-1*, but high levels of *MCL-1*, *Snail*, *Vimentin*, and *HSP47* (Figure 3F). Western blotting and qRT-PCR analysis confirmed that, knocking down PD-L1 in HEC-50 cells enhanced the expression of MCL-1, *Vimentin*, and *Snail*, and reduced the levels of *ZO-1* (Figures 3A,G). We also observed that overexpression of PD-L1 in SPAC-1-L cells led to the inhibition of EMT, featured with upregulation of *ZO-1*, and downregulation of MCL-1, *Vimentin*, and *Snail* (Figures 3A,H). Together, these data suggested that PD-L1 antagonizes EMT signaling-regulated migration and invasion in aggressive EC cells.

### PD-L1 Represses EMT by Decreasing MCL-1 Expression

**FIGURE 4:**
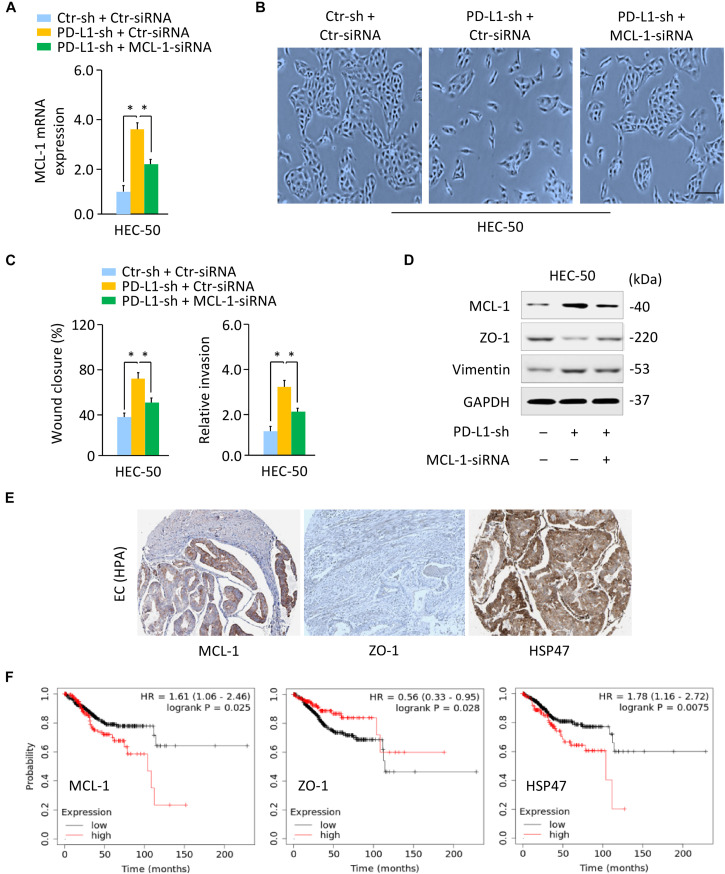
PD-L1 Represses EMT by Decreasing MCL-1 Expression. **(A)** PD-L1-silenced HEC-50 cells or control cells were transfected with (or without) MCL-1 siRNA, and *MCL-1* mRNA expression was verified using qRT-PCR analysis. **(B)** Cellular morphology of HEC-50 cells shown in panel **(A)**. Scale bar, 100 μm. **(C)** Wound-healing and invasion assays in HEC-50 cells transfected as indicated. **(D)** Western blotting analysis of the indicated proteins in HEC-50 cells transfected as indicated. **(E)** The IHC staining results were obtained from the HPA database. Immunohistochemical staining of EC tissues from the same patient showed a high MCL-1 and HSP47 expression and a low ZO-1 expression in EC tissues. **(F)** The correlation between the indicated genes and overall survival in patients with EC (KM plotter database). ^∗^*P* < 0.05.

As our previous findings pointed out the involvement of MCL-1 in EMT and the invasion of aggressive EC cells (Konno et al., 2014), we addressed whether PD-L1 regulates the EMT process and the invasion of HEC-50 cells by modulating MCL-1. We found that the silencing of MCL-1 expression with MCL-1-specific siRNA largely reversed PD-L1-sh-induced mesenchymal cellular morphology, and significantly inhibited the migratory and invasive ability that was enhanced by knockdown of PD-L1 (Figures 4A–C). Our western blotting assays further demonstrated that siRNA-induced downregulation of MCL-1 could abrogate the repression of ZO-1, and the induction of Vimentin expression by PD-L1 inhibition (Figure 4D and Supplementary Figure 3C). We then asked whether PD-L1 reduced cell growth and invasiveness through repressing MCL-1 expression in SPAC-1-L cells. Overexpression of PD-L1 in SPAC-1-L cells caused an upregulation of ZO-1 and a downregulation of Vimentin, and significantly reduced cell growth and invasion. However, restoration of MCL-1 partially abolished these effects of PD-L1 (Supplementary Figures 5A,B).

The IHC staining images for MCL-1, ZO-1, and HSP47 were obtained from the HPA database. The IHC staining of EC tissues from the same patient showed a high MCL-1 and HSP47 expression and a low ZO-1 expression in EC tissues as compared to adjacent normal tissues (Figure 4E). The correlation between *MCL-1*, *ZO-1*, and *HSP47* expression and overall survival was analyzed using the KM plotter database. Kaplan–Meier survival analysis showed that those EC patients with high levels of *MCL-1* and *HSP47* and low levels of *ZO-1* had a significantly worse overall survival rate (Figure 4F). Taken together, these data demonstrated that PD-L1 represses the EMT features of aggressive EC cells by reducing MCL-1 protein expression.

### PD-L1 Is a Direct Target of Oncogenic MiR-216a

**FIGURE 5:**
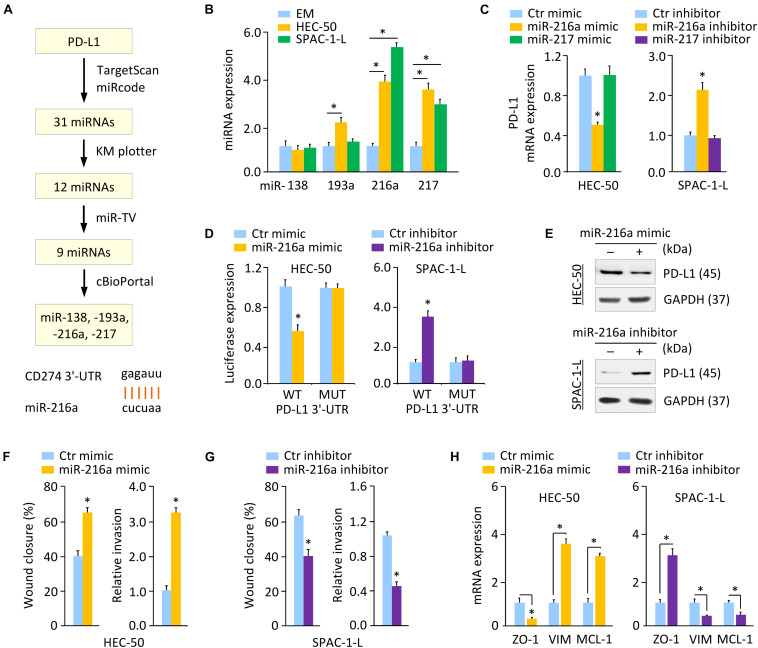
PD-L1 is a Direct Target of Oncogenic MiR-216a. **(A)** Workflow for identifying potential miRNAs that regulate the expression of PD-L1 (upper). Computational prediction of duplex formation between miR-216a with the *PD-L1* 3′-UTR sequence (bottom). **(B)** qRT-PCR analysis of the indicated miRNAs in HEC-50, SPAC-1-L, and EM cells. **(C)** *PD-L1* mRNA expression was examined using qRT-PCR assays in EC cells transfected as indicated. **(D)** Luciferase reporter assays in EC cells transfected with the wild-type (WT) or mutant (MUT) *PD-L1* 3′-UTR, along with miR-216a mimic or miR-216a inhibitor, respectively. **(E)** Western blotting analysis of PD-L1 expression in HEC-50 cells transfected with miR-216a mimic (or control mimic), and in SPAC-1-L cells transfected with miR-216a inhibitor (or control inhibitor). **(F,G)** Wound-healing and invasion assays in HEC-50 cells after overexpression of miR-216a **(F)**, or in SPAC-1-L cells after knockdown of miR-216a **(G)**. **(H)** The mRNA expression of the indicated genes was examined in HEC-50 cells after overexpression of miR-216a, and in SPAC-1-L cells after knockdown of miR-216a. VIM: Vimentin. ^∗^*P* < 0.05.

We sought to identify the potential miRNAs that directly target *PD-L1* in aggressive EC cells. A flowchart describing our screening process of miRNAs was shown in Figure 5A. We first performed a miRNA prediction analysis using the TargetScan^1^ and miRcode databases^2^. As a result, 31 miRNAs appeared simultaneously in these two databases. Next, screening of the KM plotter database enabled us to find 12 miRNAs, whose expression is associated with a worse prognosis in EC (Supplementary Figure 6). Analysis of the TCGA EC datasets through the miR-TV database (Pan and Lin, 2020) showed that, nine miRNAs exhibited a significantly higher expression in EC tissues compared with normal tissues (Supplementary Figure 7A). The expression of these nine miRNAs in TCGA EC tissues was examined using the cBioPortal database (Gao et al., 2013). Four miRNAs, including miR-138, miR-193a, miR-216a, and miR-217, were amplified in patients with EC (Supplementary Figure 7B).

Using qRT-PCR assays, we validated that, of the four miRNAs, only miR-216a and miR-217 were consistently upregulated in aggressive EC cells as compared to EM cells (Figure 5B and Supplementary Figure 7C). Thus, we determined that miR-216a and miR-217 were likely candidates for further investigation. Compared to the respective controls, the mRNA expression of *PD-L1* decreased markedly when miR-216a (but not miR-217) was overexpressed in HEC-50 cells (Figure 5C). Conversely, the expression of PD-L1 increased significantly after miR-216a (but not miR-217) was inhibited in SPAC-1-L cells (Figure 5C).

Luciferase reporter assays were further conducted by employing a luciferase reporter vector containing the *PD-L1* 3′-UTR sequence flanking the putative binding site of miR-216a. Mutations in the putative binding sites were created as controls. The overexpression of miR-216a significantly reduced the luciferase activities of WT *PD-L1* 3′-UTR in HEC-50 cells, and the inhibition of miR-216a increased the luciferase activities of WT *PD-L1* 3′-UTR in SPAC-1-L cells (Figure 5D). The transfection with miR-216a mimic or miR-216a inhibitor into EC cells showed no significant effects on the luciferase activities of MUT *PD-L1* 3′-UTR (Figure 5D). These results were validated by western blotting assays showing that overexpression of miR-216a suppressed the protein expression of PD-L1, whereas inhibition of miR-216a increased the levels of PD-L1 in EC cells (Figure 5E and Supplementary Figure 3D). These data suggested that PD-L1 is a direct target of miR-216a in aggressive EC cells.

To determine the role of miR-216a in aggressive EC cells, we overexpressed miR-216a in HEC-50 cells that have a low endogenous expression of miR-216a, and also transfected miR-216a inhibitor into SPAC-1-L cells expressing high levels of miR-216a. Overexpression of miR-216a significantly induced the migration and invasion of HEC-50 cells (Figure 5F). Cell migration and invasion were significantly reduced in SPAC-1-L cells following knockdown of miR-216a (Figure 5G). Consistently, the mRNA expression of *MCL-1* and *Vimentin* was upregulated, whereas the levels of *ZO-1* were decreased, when miR-216a was overexpressed (Figure 5H). In contrast, miR-216a-silenced SPAC-1-L cells showed decreased *MCL-1* and Vimentin, and increased *ZO-1* expression (Figure 5H). These results demonstrated that PD-L1 is a direct target of oncogenic miR-216a in aggressive EC cells.

### MEG3 Acts as an Upstream Regulator of MiR-216a and PD-L1

**FIGURE 6:**
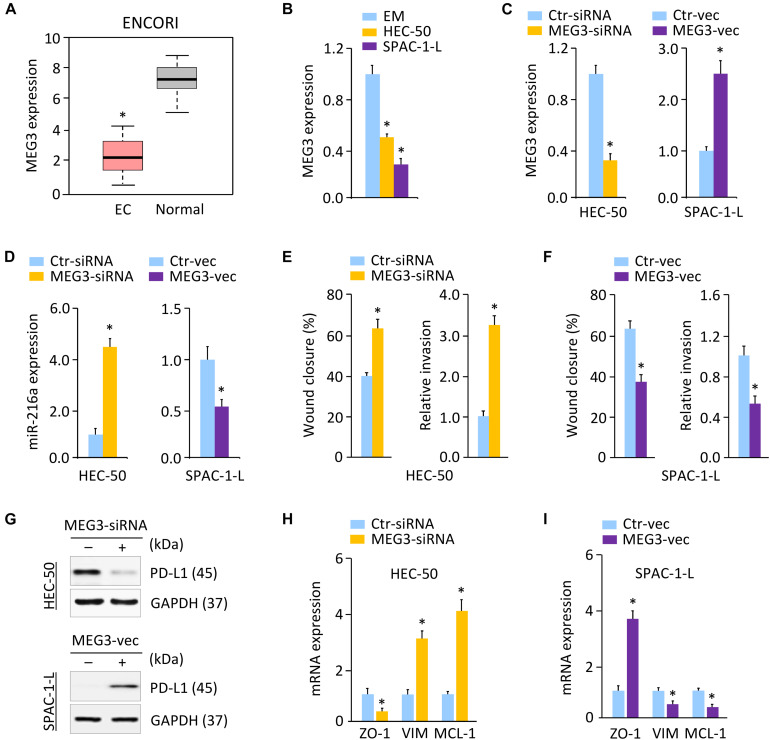
MEG3 Acts as an Upstream Regulator of MiR-216a and PD-L1. **(A)** MEG3 expression in TCGA EC tissues and normal tissues (ENCORI database). **(B)** qRT-PCR analysis of MEG3 expression in HEC-50, SPAC-1-L and EM cells. **(C)** MEG3 levels in HEC-50 cells transfected with MEG3 siRNA (or control siRNA), and in SPAC-1-L cells transfected with MEG3 expression vector (or control vector). **(D)** miR-216a expression was measured in HEC-50 cells transfected with MEG3 siRNA (or control siRNA), and in SPAC-1-L cells transfected with MEG3 expression vector (or control vector). **(E)** Wound-healing and invasion assays in HEC-50 cells transfected as indicated. **(F)** Wound-healing and invasion assays in SPAC-1-L cells transfected as indicated. **(G)** Examination of PD-L1 expression in HEC-50 cells following knockdown of MEG3, and in SPAC-1-L cells following overexpression of MEG3, using western blotting assays. **(H,I)** The mRNA expression of the indicated genes in HEC-50 cells following knockdown of MEG3, and in SPAC-1-L cells following overexpression of MEG3. VIM: Vimentin. ^∗^*P* < 0.05.

Long non-coding RNAs play key roles in human cancers, including aggressive EC (Dong et al., 2019b). Several lncRNAs, such as NEAT1 and MEG3, were reported to regulate cancer tumorigenesis and metastasis through their interactions with DNA, RNA, and proteins (Dong et al., 2018b, Dong et al., 2019a, b). To elucidate the mechanisms governing PD-L1 expression, we predicted the lncRNAs that may interact with miR-216a, by performing a sequence alignment analysis through the ENCORI and LncBase Predicted v.2 databases (Paraskevopoulou et al., 2016). To increase the prediction accuracy, we extracted the overlapping part of the prediction results across these two databases, and identified 26 candidate lncRNAs (data not shown). Analysis of the TCGA EC database in the GEPIA database (Tang et al., 2017) further showed that, high expression of three candidate lncRNAs (including MEG3, RPARP-AS1, and SNHG5) was a favorable prognostic marker for EC (Supplementary Figure 8A). According to the results from the ENCORI database, only MEG3 (but not RPARP-AS1 and SNHG5) exhibited significantly lower expression in TCGA EC tissues as compared to the normal tissues (Figure 6A). Comparison of the expression of MEG3 in three aggressive EC cell lines (HEC-1, HEC-50, and KLE) via the Expression Atlas database revealed that, similar to *PD-L1* and *ZO-1*, the expression level of MEG3 was also low in these cells (Figure 3F). Using qRT-PCR analysis, we discovered that the levels of MEG3 were significantly downregulated in HEC-50 and SPAC-1-L cells compared with EM cells (Figure 6B), suggesting a potential tumor-suppressor role for MEG3 in aggressive EC cells.

Long non-coding RNAs were considered to act as sponges for miRNAs and inhibit their expression (Dong et al., 2018b, Dong et al., 2019a). Given that MEG3 acts as a tumor suppressor to regulate EC progression by functioning as a competing endogenous RNA (Dong et al., 2019b), and that MEG3 contains the predicted miR-216a-binding site (Supplementary Figure 8B), we speculated that MEG3 might positively regulate the levels of PD-L1 by decreasing miR-216a expression in aggressive EC cells. In line with this notion, knocking down MEG3 promoted but the ectopic expression of MEG3 inhibited miR-216a expression in aggressive EC cells (Figures 6C,D). The role of MEG3 in suppressing cell migration was confirmed by wound-healing assays in HEC-50 and SPAC-1-L cells (Figure 6E,F). Consistent with these results, we found that knockdown of MEG3 inhibited the protein expression of PD-L1 compared to control cells, while overexpression of MEG3 increased the expression of PD-L1 in EC cells (Figure 6G and Supplementary Figure 3E). Our qRT-PCR experiments showed that transfection with MEG3-specific siRNA downregulated *ZO-1*, and upregulated *Vimentin* and *MCL-1* in HEC-50 cells (Figure 6H). However, overexpression of MEG3 had the opposite effects in SPAC-1-L cells (Figure 6I). As expected, the inhibition of MEG3 in SPAC-1-L cells significantly induced cell proliferation and invasion, whereas overexpression of MEG3 could significantly reduce the proliferation and invasion of HEC-50 cells (Supplementary Figures 9A–C). Taken together, these data supported the possibility that MEG3 negatively regulates the expression of miR-216a, thus abolishing the miR-216a-induced repressive effects on the *PD-L1* 3′-UTR.

## Discussion

**FIGURE 7:**
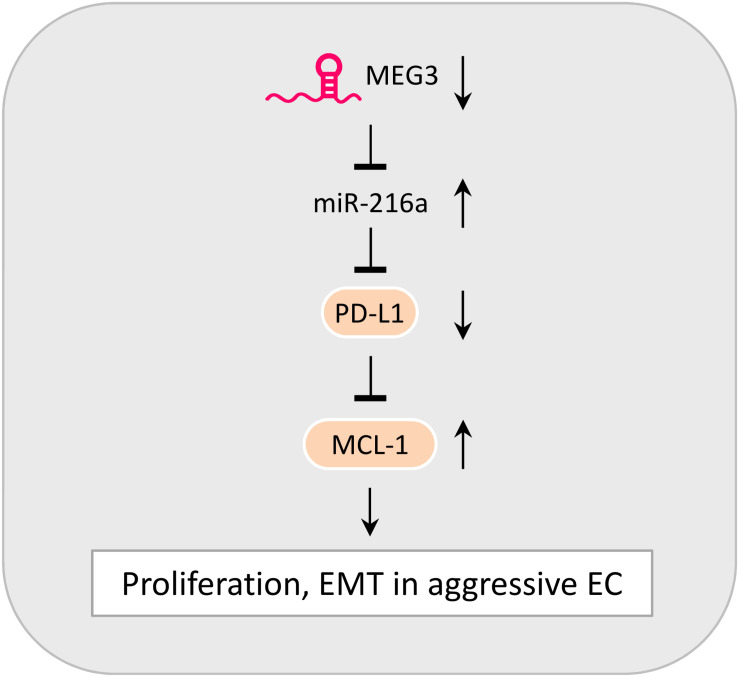
Model Summarizing the Role of MEG3, MiR-216a, PD-L1, and MCL-1 in Controlling the Proliferative and Invasive Potential of aggressive EC cells. The downregulation of MEG3 causes an elevation of miR-216a expression. By targeting the 3′-UTR of *PD-L1* mRNA, miR-216a suppresses PD-L1 expression. PD-L1 serves as a tumor-suppressor to inhibit the proliferation, EMT and invasive potential of aggressive EC cells via repressing MCL-1 expression.

The incidence rate of aggressive EC has been rapidly increasing in the United States and Japan (Yamagami et al., 2017; Clarke et al., 2019). The highly metastatic and often treatment-refractory nature of aggressive EC correlates with poor patient survival (Gaber et al., 2016). A better understanding of the mechanisms behind the tumorigenesis and metastasis of aggressive EC is urgently needed to improve the early diagnosis and effective treatment of this cancer. EMT induction and immune evasion have been demonstrated to promote cancer development (Valastyan and Weinberg, 2011; Gonzalez et al., 2018). Although recent studies have linked EMT processes to immune escape (Terry et al., 2017; Dong et al., 2018c), little is known about the functional significance of PD-L1 in aggressive EC cells. Our study demonstrates a new tumor-suppressor role of PD-L1 in repressing the proliferation and EMT-associated migration and invasion in aggressive EC cells, and reveals that the downregulation of MEG3 and induction of its downstream effector miR-216a is likely a novel mechanism underlying the downregulation of PD-L1 observed in EC tissues (Figure 7).

The expression pattern of PD-L1 and its prognostic value in EC is seemingly controversial in the literature (Mo et al., 2016; Li et al., 2018; Marinelli et al., 2019; Engerud et al., 2020). These conflicting results may be attributed to either technical reasons or different clinical features of the analyzed samples (Marinelli et al., 2019). By employing a validated antibody that exhibited high sensitivity and high specificity (Dong et al., 2018b; Nagy et al., 2018), we found that, compared with normal endometrium samples, the protein expression of PD-L1 was frequently lost in EC tissues. In line with previous reports describing an inverse correlation between the levels of PD-L1 and the degree of tumor malignancy in human EC (Engerud et al., 2020; Zhang et al., 2020), we found that higher protein expression of PD-L1 seems to correlate with younger patient age, low-grade disease, early stage tumors, smaller tumor size, or superficial myometrial invasion. Future studies with a larger sample size are necessary to validate our current data.

Moreover, previous research has shown that, in patients with metastatic melanoma (Taube et al., 2012), colorectal cancer (Droeser et al., 2013), Merkel-cell carcinoma (Lipson et al., 2013), and EC (Liu et al., 2015), higher PD-L1 expression was correlated with improved overall survival rates, suggesting that high PD-L1 expression may be a favorable prognostic marker in several types of cancer. Consistent with these reports, our data suggest that PD-L1 loss can identify EC patients with a worse probability of survival, and that lower expression of PD-L1 was particularly associated with shorter overall survival in high-grade ECs, indicating that a PD-L1-negative expression signature might be an indicator of poor prognosis in ECs with aggressive behaviors.

Programmed death-ligand-1 exhibits pro-tumor effects via various mechanisms (Clark et al., 2016; Gupta et al., 2016; Gato-Cañas et al., 2017). Tumor cell-intrinsic PD-L1 promotes melanoma tumorigenesis *in vivo* through activating the mTOR signaling (Clark et al., 2016). In murine melanoma cells, PD-L1 confers resistance to interferon cytotoxicity and accelerates tumor progression via a STAT3/caspase-7-dependent pathway (Gato-Cañas et al., 2017). PD-L1 was also shown to enhance tumor sphere formation of ovarian cancer cells possibly by increasing SOX2 expression (Gupta et al., 2016). The molecular link between high PD-L1 expression and EMT in cancer cells has been noticed (Chen et al., 2017; Qiu et al., 2018). In glioblastoma multiforme cells, PD-L1 activates the EMT process by upregulating Slug, β-catenin and Vimentin and by downregulating E-cadherin, via activation of the RAS/MEK/ERK signaling (Qiu et al., 2018). Overexpression of PD-L1 enhances the levels of mesenchymal genes (ZEB1, N-cadherin, and Vimentin), and contributes to the EMT phenotypes of esophageal cancer cells (Chen et al., 2017). However, silencing PD-L1 in cholangiocarcinoma cells by shRNA can increase tumorigenic potential and ALDH activity (Tamai et al., 2014). Furthermore, overexpression of PD-L1 significantly decreases the activities of PI3K/AKT and RAS/MEK/ERK pathways, leading to the suppression of lung cancer cell growth *in vitro* and in xenografts (Wang X. et al., 2020), providing evidence for the tumor suppressive role of PD-L1 in specific tumor type. Our study revealed that, via repression of MCL-1, PD-L1 could induce the expression of ZO-1, while suppressing the expression of Vimentin. These results showed that tumor cell-intrinsic PD-L1 has tumor-suppressive functions in aggressive EC cells, at least through its negative modulation of EMT. Thus, the silencing of PD-L1 may underline the molecular mechanisms for inducing and maintaining the mesenchymal state of aggressive EC cells.

Activation of the PI3K/AKT axis is known to be a central feature of EMT in numerous cancers (Dong et al., 2014). In addition, aberrant activation of the RAS/MEK/ERK pathway in human cancer cells allows them to undergo EMT via the upregulation of Snail (Tripathi and Garg, 2018). MCL-1 is an important downstream effector of PI3K/AKT and RAS/MEK/ERK signaling (Xiang et al., 2018). In this study, we demonstrated that the upregulation of MCL-1 caused by PD-L1 silencing contributes to EMT in aggressive EC cells. Further investigation will be required to determine whether PD-L1 represses the expression of MCL-1 to attenuate EMT in aggressive EC cells, through the PI3K/AKT and RAS/MEK/ERK signaling pathways.

Since PD-L1 has a critical role in suppressing anti-tumor immunity, cancer immunotherapy (in particular antibodies that block the PD-1/PD-L1 interaction) was considered to be a revolution in cancer treatment (Postow et al., 2015), and has generated clinical benefit in a subset of patients with EC (Green et al., 2020). Recently, alternative strategies (such as combination therapies with chemotherapy and siRNA against PD-L1 (Yoo et al., 2019)), have been proposed. A nanocarrier-aided delivery of PD-L1 siRNA, together with gemcitabine, resulted in a significant reduction in pancreatic cancer growth (Yoo et al., 2019). However, our cell functional study revealed that tumor cell-intrinsic PD-L1 plays an anti-tumor role in multiple aggressive EC cell lines, and downregulation of PD-L1 is sufficient to stimulate the EMT features and cell invasion in an MCL-1-dependent manner. Thus, designing therapeutic strategies aimed at knocking down PD-L1 expression in aggressive EC may lead to unexpected outcomes, possibly by accelerating EMT and metastasis. Future research is needed to explore this possibility.

Although a tumor-suppressive role for miR-216a has been reported (Zhang et al., 2017), this miRNA was identified as an oncogenic miRNA in many cancers, including EC (Wang Q.A. et al., 2020), ovarian cancer (Liu et al., 2017), hepatocellular carcinoma (Xia et al., 2013), and renal cell carcinoma (Chen et al., 2018). The direct target genes of miR-216a include *PTEN* in EC (Wang Q.A. et al., 2020), and *PTEN* and *SMAD7* in hepatocellular carcinoma (Xia et al., 2013). We have validated that, by directly targeting *PD-L1* 3′-UTR, miR-216a decreases PD-L1 levels to promote EMT and cell invasion in aggressive EC cells. It would be interesting to further determine the downstream targets of miR-216a and the clinical significance of miR-216a-regulated pathways in this clinically important subtype of EC.

Prior studies demonstrated that MEG3 is located at human chromosome 14q32.3 and is a novel tumor-suppressor lncRNA in many tumors, including EC (He et al., 2017; Dong et al., 2019b). Restoring the expression of MEG3 could suppress cancer initiation, progression, metastasis and chemoresistance (He et al., 2017). MEG3 levels were downregulated in EC tissues compared with that in adjacent non-tumor tissues, and EC patients with low MEG3 expression exhibited shorter overall survival compared with patients with high expression levels (Dong et al., 2019b). Here, we defined an anti-cancer function of MEG3 through the regulation of EMT in aggressive EC cells. Multiple molecular mechanisms, including gene deletion and promoter hypermethylation, contribute to the loss of MEG3 expression in tumor cells (He et al., 2017). Further studies are necessary to unravel the regulatory mechanisms of MEG3 expression in EC development.

## Conclusion

In summary, our findings reveal that PD-L1 has a tumor cell-intrinsic role in suppressing the proliferation and EMT features of aggressive EC cells. This study identifies MEG3 and miR-216a as critical upstream regulators of PD-L1, thus providing a previously unreported mechanism responsible for PD-L1 dysregulation in aggressive EC cells.

## Data Availability Statement

The original contributions presented in the study are included in the article/Supplementary Material, further inquiries can be directed to the corresponding author/s.

## Ethics Statement

The studies involving human participants were reviewed and approved by The Research Medical Ethics Committee of Sun Yat-sen University Cancer Center. The patients/participants provided their written informed consent to participate in this study.

## Author Contributions

DX, PD, and YK designed the experiments. DX, PD, YX, and RC performed the experiments. JY, YK, KI, and HW analyzed the data. DX and PD wrote the manuscript. All authors read and approved the final manuscript.

## Conflict of Interest

The authors declare that the research was conducted in the absence of any commercial or financial relationships that could be construed as a potential conflict of interest.

## Footnotes

1 http://www.targetscan.org/vert_72/

2 http://www.mircode.org/
