# Supplementary material for: PD-L1 Is a Tumor Suppressor in Aggressive Endometrial Cancer Cells and Its Expression Is Regulated by miR-216a and lncRNA MEG3
Source: Front Cell Dev Biol. 2020 Dec 9;8:598205. doi: 10.3389/fcell.2020.598205 (PMC7755603; doi:10.3389/fcell.2020.598205)

Supplementary Material

PD-L1 is a tumor suppressor in aggressive endometrial cancer cells and its expression is regulated by miR-216a and lncRNA MEG3

Daozhi Xu ^1, †^, Peixin Dong ^1, †,^ *, Ying Xiong ^2, †^, Rui Chen ^2^, Yosuke Konno ^1,^ *, Kei Ihira ^1^, Junming Yue ^3, 4^, and Hidemichi Watari ^1^

1. Department of Obstetrics and Gynecology, Hokkaido University School of Medicine, Hokkaido University, Sapporo 060-8638, Japan; xudaozhi87@yahoo.co.jp (D.X.); konsuke013@gmail.com (Y.K.); ihey0610@huhp.hokudai.ac.jp (K.I.); watarih@med.hokudai.ac.jp (H.W.)
2. Department of Gynecology, State Key Laboratory of Oncology in South China, Sun Yat-sen University Cancer Center, Guangzhou 510060, China; tdken999@163.com (Y.X.); 455327605@qq.com (R.C.)
3. Department of Pathology and Laboratory Medicine, University of Tennessee Health Science Center, Memphis, TN 38163, USA; [jyue@uthsc.edu](mailto:jyue@uthsc.edu) (J.Y.)
4. Center for Cancer Research, University of Tennessee Health Science Center, Memphis, TN 38163, USA

^†^ Contributed equally

*  **Correspondence:** Peixin Dong (dpx1cn@gmail.com) or Yosuke Konno

([konsuke013@gmail.com](mailto:konsuke013@gmail.com))

Supplementary Figure 1

# High PD-L1 represents a favorable factor for the prognosis of EC patients with high-grade tumors

The probability of overall survival in EC patients with grade 2 (left) or grade 3 (right) tumors, stratified by high or low PD-L1 levels (KM plotter database).


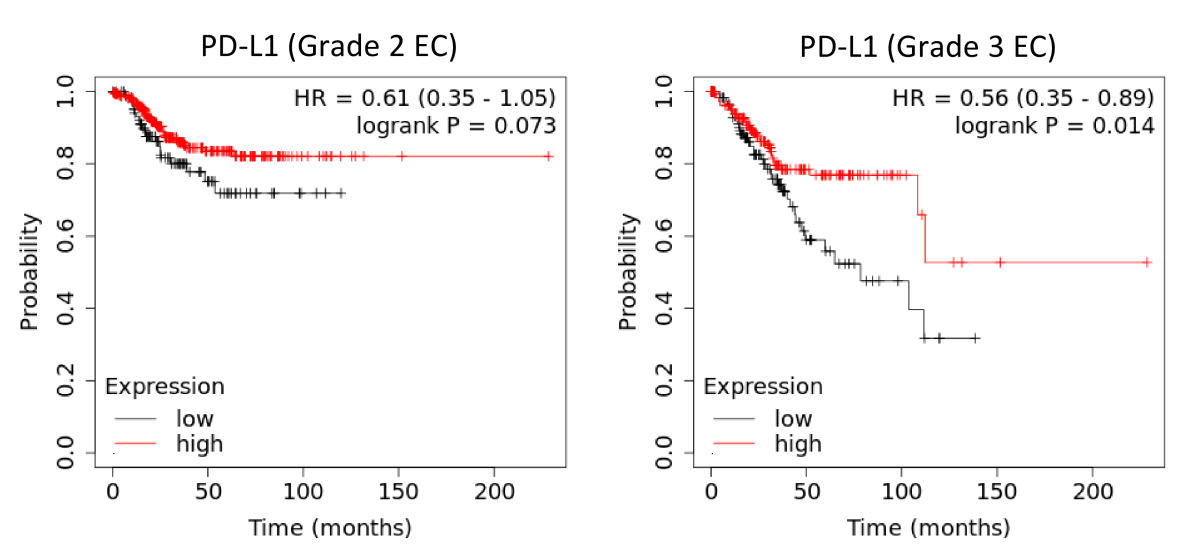


Supplementary Figure 2

**The association between PD-L1 expression and clinicopathological factors of human EC**

The pie charts representing IHC results for the proportion of EC tissues with PD-L1-low vs. PD-L1-high expression, stratified by patient age, tumor grade, tumor stage, tumor diameter, or myometrial invasion.


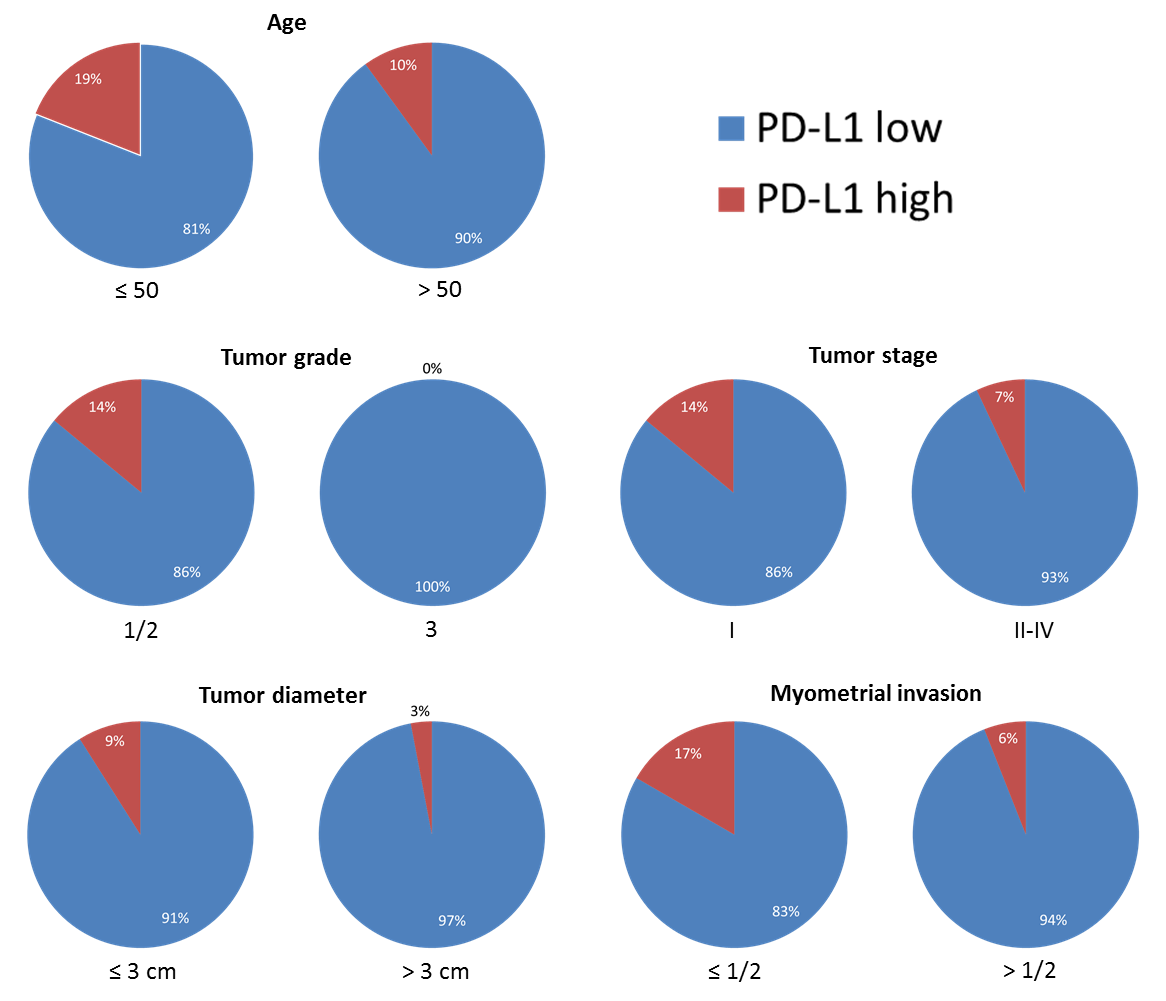


Supplementary Figure 3

Quantification of the indicated protein expression normalized to GAPDH.

**P* < 0.05.


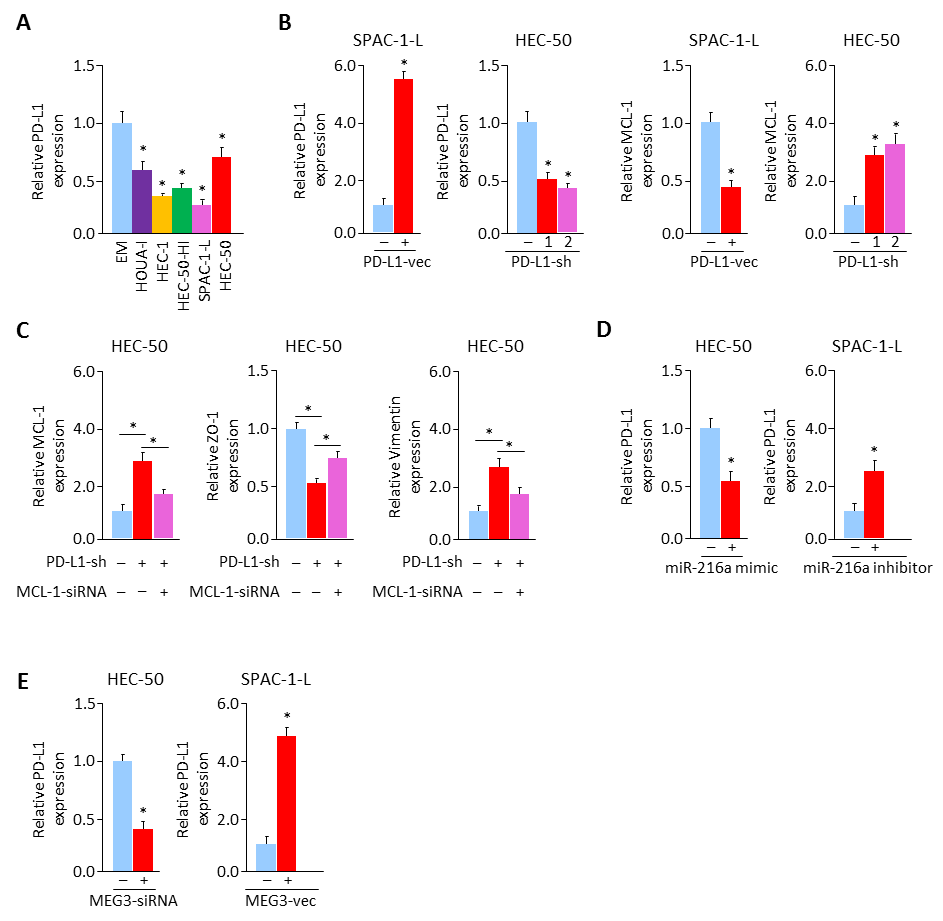


Supplementary Figure 4

**PD-L1 reduces the proliferation and invasion of EC cells**

(A) Left panel: western blotting analysis of PD-L1 expression in PD-L1-silenced SPAC-1-L cells, and in HEC-50 cells overexpressing PD-L1. Right panel: quantitative analysis of the western blots shown in A, respectively (normalized to GAPDH). B) Proliferation and invasion assays in SPAC-1-L cells after knockdown of PD-L1. (C) Proliferation and invasion assays in HEC-50 cells after overexpression of PD-L1. **P* < 0.05.


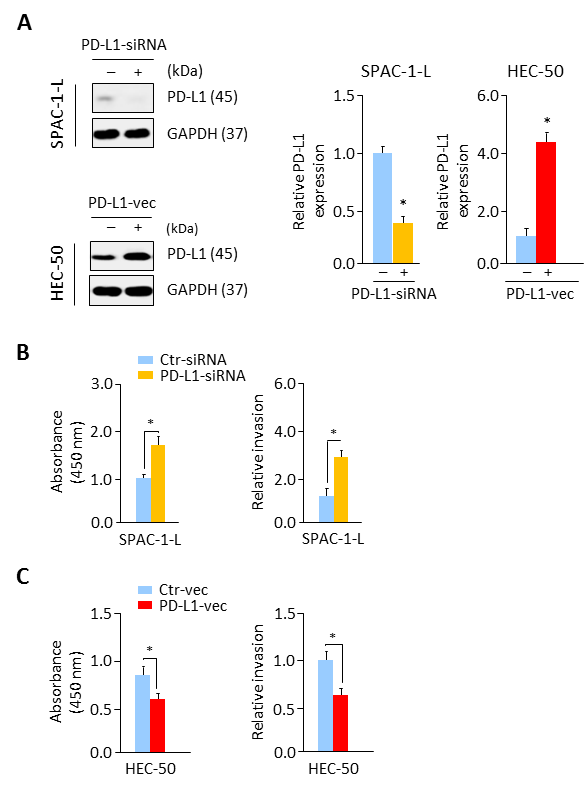


Supplementary Figure 5

**Overexpression of PD-L1 reduces the proliferation and EMT of SPAC-1-L cells via suppressing MCL-1 levels**

(**A**) SPAC-1-L cells overexpressing PD-L1 or the control cells were transfected with MCL-1-vec or Ctr-vec, respectively. Left panel: the protein levels of MCL-1, ZO-1, and Vimentin were assessed using Western blotting analysis. Right panel: quantitative analysis of the western blots shown in **A**, respectively (normalized to GAPDH). (**B**) CCK-8 and invasion assays were performed to evaluate cell proliferation and invasiveness. **P* < 0.05.


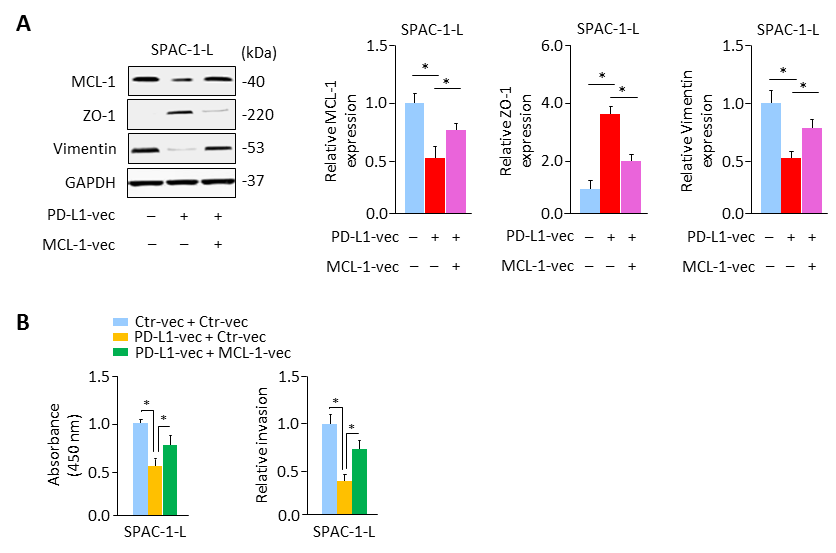


Supplementary Figure 6

**The prognostic significance of miRNAs in EC**

The correlation between the expression of indicated miRNAs and overall survival in patients with EC was analyzed using the KM plotter database.


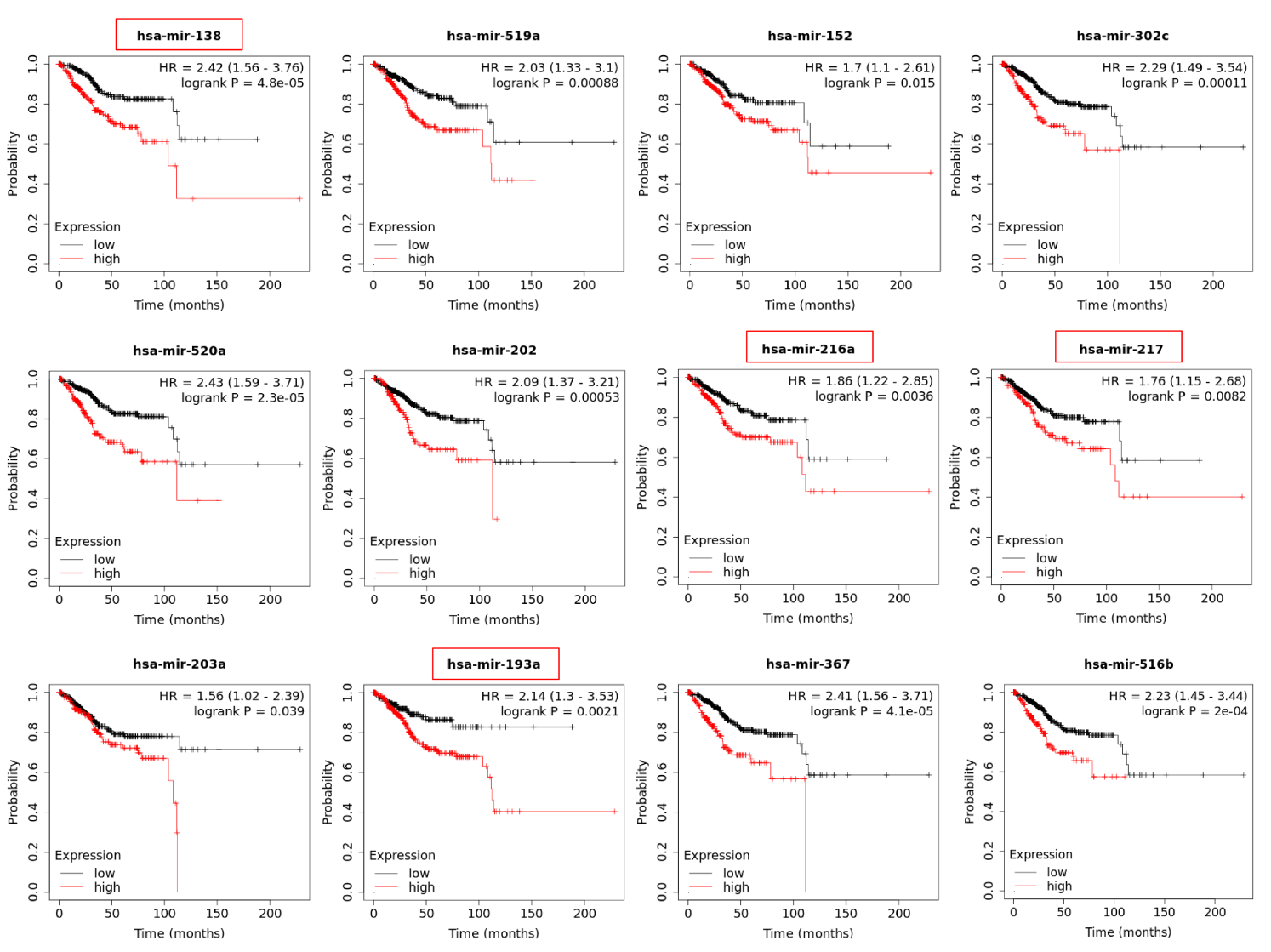


Supplementary Figure 7

**The expression profile of miRNAs in EC**

(**A**) MiRNA expression in TCGA EC samples and normal samples was analyzed using the miR-TV database. (**B**) Oncoprint plot showing an overview of the genetic alterations in four miRNAs from TCGA EC samples (cBioPortal database). (**C**) qRT-PCR analysis of miR-216a expression in HOUA-I, HEC-1, HEC-50-HI and EM cells. **P* < 0.05.


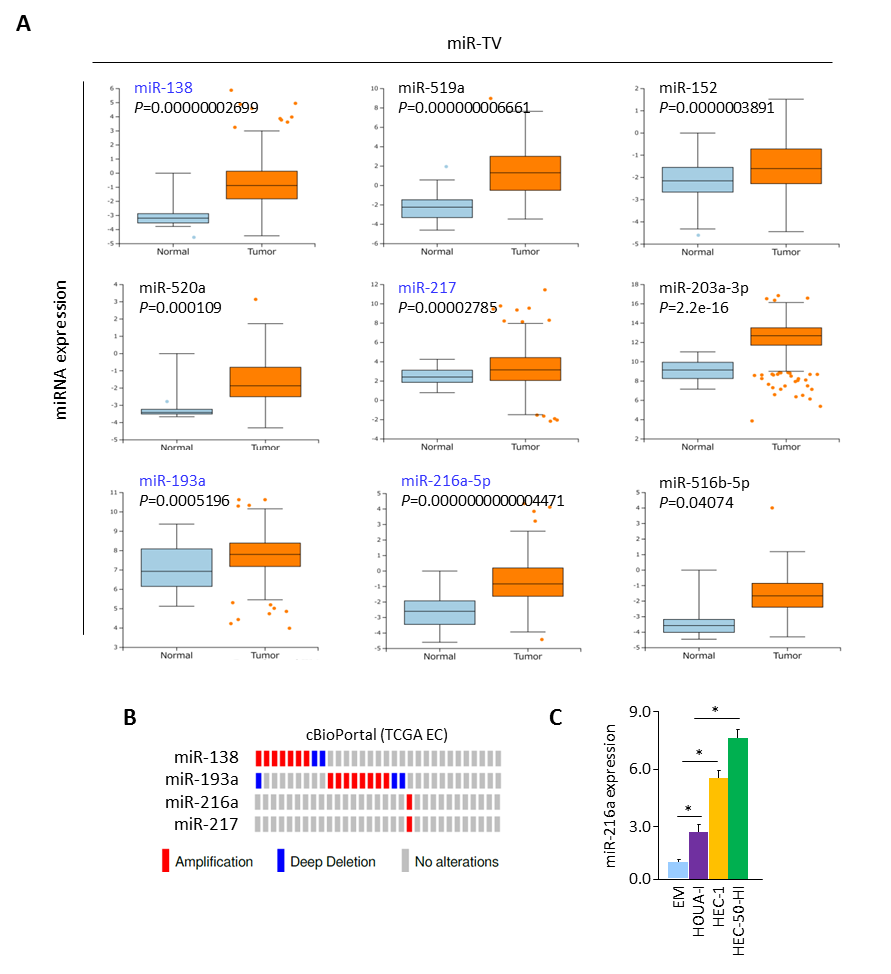


Supplementary Figure 8

**The prognostic value of candidate long non-coding RNAs in EC**

(**A**) The correlation between the expression of indicated long non-coding RNAs and overall survival in patients with EC was analyzed using the GEPIA database. (**B**) Computational prediction of duplex formation between miR-216a with the MEG3 sequence (ENCORI database).


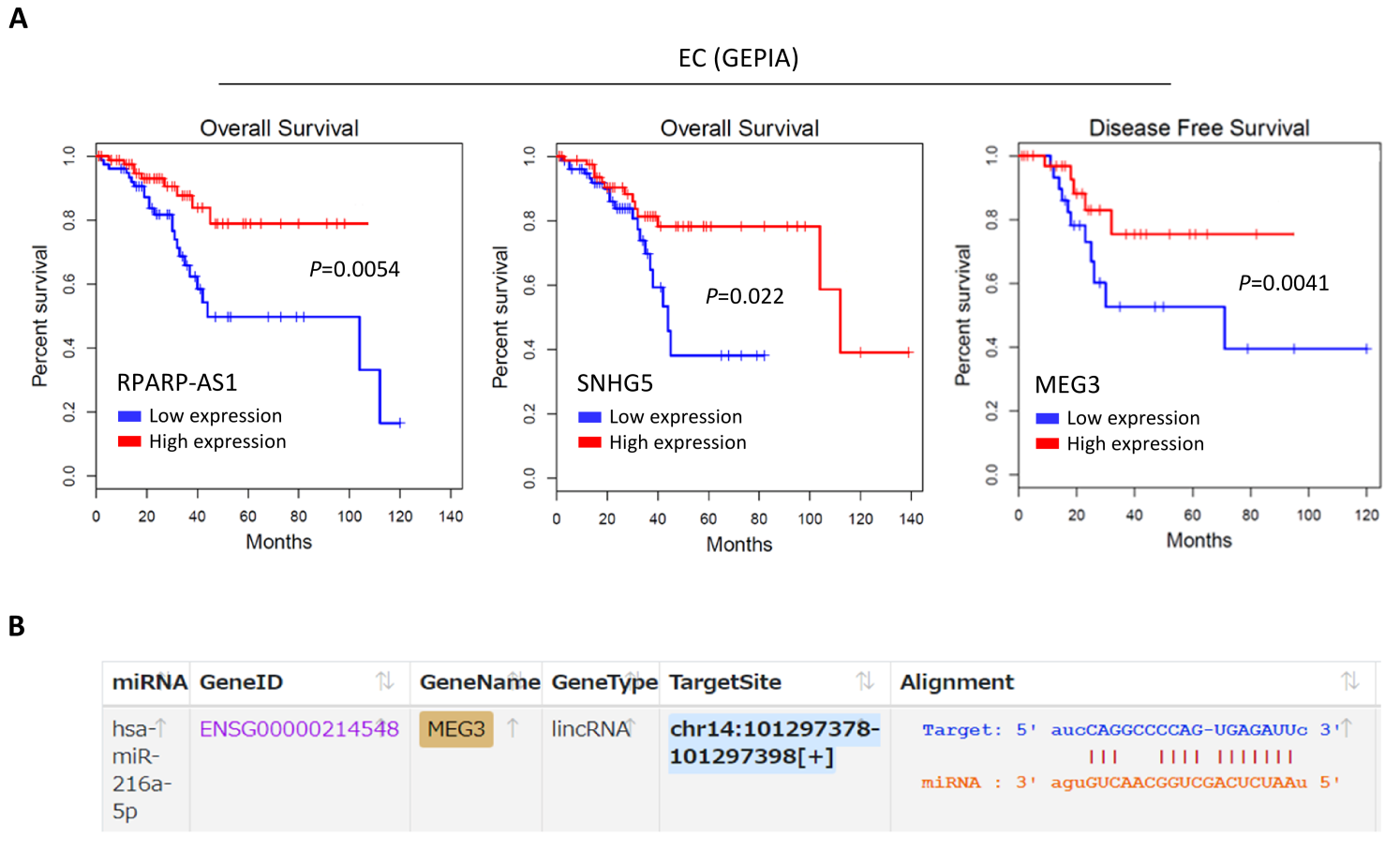


Supplementary Figure 9

**MEG3 represses the proliferation and invasion of EC cells**

(A) qRT-PCR analysis of MEG3 expression in HEC-50 cells overexpressing MEG3, and in MEG3-silenced SPAC-1-L cells. (B) Proliferation and invasion assays in SPAC-1-L cells after knockdown of MEG3. (C) Proliferation and invasion assays in HEC-50 cells after overexpression of MEG3. **P* < 0.05.


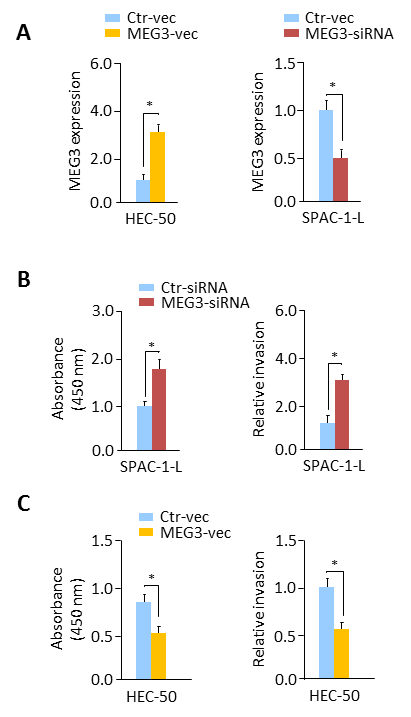

Supplement: Supplementary file 1 [file Data_Sheet_1.docx]
